# Polysaccharide Structures and Their Hypocholesterolemic Potential

**DOI:** 10.3390/molecules26154559

**Published:** 2021-07-28

**Authors:** Inês M. V. Silva, Fernanda Machado, Maria João Moreno, Cláudia Nunes, Manuel A. Coimbra, Filipe Coreta-Gomes

**Affiliations:** 1 Coimbra Chemistry Center, University of Coimbra, Rua Larga Largo D. Dinis, 3004-535 Coimbra, Portugal; imvs@qui.uc.pt (I.M.V.S.); mmoreno@ci.uc.pt (M.J.M.); 2 LAQV-REQUIMTE, Chemistry Department, University of Aveiro, 3810-193 Aveiro, Portugal; fernandamachado@ua.pt (F.M.); claudianunes@ua.pt (C.N.); mac@ua.pt (M.A.C.); 3 Chemistry Department, Faculty of Science and Technology, University of Coimbra, Rua Larga Largo D. Dinis, 3004-535 Coimbra, Portugal; 4 CICECO—Aveiro Institute of Materials, Chemistry Department, University of Aveiro, 3810-193 Aveiro, Portugal

**Keywords:** polysaccharides, chitosan, β-glucans, cholesterol homeostasis, viscosity, bile salt sequestration, microbiota, hypocholesterolemic ingredients, fiber

## Abstract

Several classes of polysaccharides have been described to have hypocholesterolemic potential, namely cholesterol bioaccessibility and bioavailability. This review will highlight the main mechanisms by which polysaccharides are known to affect cholesterol homeostasis at the intestine, namely the effect (i) of polysaccharide viscosity and its influence on cholesterol bioaccessibility; (ii) on bile salt sequestration and its dependence on the structural diversity of polysaccharides; (iii) of bio-transformations of polysaccharides and bile salts by the gut microbiota. Different quantitative structure–hypocholesterolemic activity relationships have been explored depending on the mechanism involved, and these were based on polysaccharide physicochemical properties, such as sugar composition and ramification degree, linkage type, size/molecular weight, and charge. The information gathered will support the rationalization of polysaccharides’ effect on cholesterol homeostasis and highlight predictive rules towards the development of customized hypocholesterolemic functional food.

## 1. Introduction

Cholesterol related diseases are responsible for high levels of death and impairment worldwide [1]. Although there are synthetic drugs available to control cholesterol blood levels, which can promote adverse side effects [1,2,3], natural origin hypocholesterolemic food ingredients can act synergistically, aiding their use. Polysaccharide-based ingredients such as β-glucans are currently used as hypocholesterolemic food ingredients, with health claims accepted by European Food Safety Agency (EFSA) and Food and Drug Administration (FDA) [4]. Industry and scientific players continue to seek the development of selective, effective, and low-cost compounds with a wide range of applications in food matrices. The research on the development of new polysaccharides with hypocholesterolemic potential is very active, being focused mainly on finding new sources of polysaccharides with cholesterol reducing properties, based on new food products and customizing potential hypocholesterolemic polysaccharides by physicochemical and biochemical methods.

**Figure 1:**
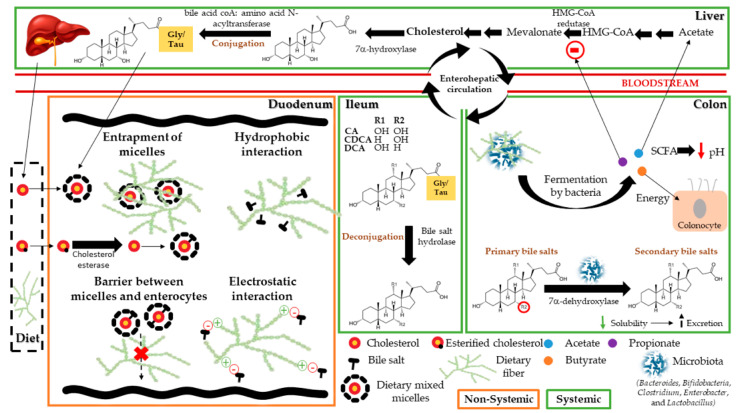
Schematic representation of cholesterol homeostasis steps.

Cholesterol present in the human body has two main sources, namely the diet and that endogenously produced at the liver, accounting for one and two thirds, respectively, in a total daily of 1800 mg [5]. Dietary cholesterol may occur as free or esterified cholesterol. Here, the latter need to be deesterified by cholesterol esterase, in order to be able to be absorbed [6]. Prior to absorption, both cholesterol sources are emulsified by the action of bile salts (BS), fatty acids, and phospholipids among others, forming different dietary mixed aggregates, suffering different disassembly processes. These reassemble into mixed micelles prior to absorption [7,8]. Polysaccharides can affect cholesterol homeostasis, depending on their intrinsic physicochemical characteristics, such as viscosity, molecular weight, solubility, and charge among others, as well as sugar composition, ramification, and sugar linkages. The effects are mostly through BS sequestration and/or through active moieties produced by polysaccharide fermentation by gut microbiota [9,10,11,12,13,14]. Regarding dietary cholesterol, the inhibition of cholesterol esterase by polysaccharides in intestinal lumen has been described to affect cholesterol bioavailability [6]. It is therefore of paramount importance to understand the mechanisms of action by which polysaccharides affect cholesterol bioaccessibility, but also how they can affect cholesterol biosynthesis, as well as possible synergies between the different known hypocholesterolemic mechanisms. One of the most described mechanisms that affects cholesterol homeostasis concerns the effect of polysaccharides on the viscosity of the intestinal lumen content. This rise of viscosity affects the diffusion of dietary mixed micelles loaded with cholesterol towards the intestinal epithelium membrane, limiting cholesterol bioavailability [13,14]. Moreover, the sequestration of BS by polysaccharides is relevant as a hypocholesterolemic strategy, because the lower amount of bile salts in solution reduces the emulsification power towards cholesterol, leading to its precipitation and consequent excretion into the feces [15]. The higher excretion of BS also leads to a lower extent of BS enterohepatic recirculation at ileum, which in turn increases the conversion of endogenous cholesterol produced at the liver to primary bile salts, namely cholic and chenodeoxycholic acids conjugated with glycine [16]. Another important mechanism regarding the hypocholesterolemic potential of polysaccharides concerns their fermentation by microbiota and their resulting metabolites. This generates short chain fatty acids (SCFA), which interfere with cholesterol biosynthesis, and convert primary into secondary BS, relevant for cholesterol emulsification [17,18,19]. Figure 1 shows a schematic description of cholesterol homeostasis steps, focusing on non-systemic hypocholesterolemic strategies affecting cholesterol bioaccessibility at intestinal lumen and systemic strategies which are related to the effect of bioavailable metabolites towards the blood stream and their influence on the endogenous cholesterol production at liver.

This work addresses the structure of hypocholesterolemic polysaccharides from both in vitro and in vivo experiments and, whenever possible, clarifies their possible mechanisms of action. The work is divided into three main sections: (1) Polysaccharide viscosity and its influence on cholesterol bioaccessibility; (2) Bile salts sequestration and its dependence on the structural diversity of polysaccharides; (3) Microbiota bio-transformations of polysaccharides and BS: hypocholesterolemic implications.

## 2. Polysaccharides Viscosity and Its Influence on Cholesterol Bioaccessibility

Cholesterol solubilization at intestinal lumen by mixed micelles composed of BS and dietary components is a mandatory process for cholesterol to reach the intestinal epithelium, where it may be absorbed by simple diffusion or through the action of cholesterol transporter (e.g., Niemann Pick C1 L1) [5]. Polysaccharides, usually present in the human diet, have an impact on micelle diffusion and therefore on cholesterol bioaccessibility. They can affect intestinal lumen viscosity due to their ability to thicken or form gels potentiated by physical entanglements, dependent on the monomeric units that compose the polysaccharide. 

**Figure 2:**
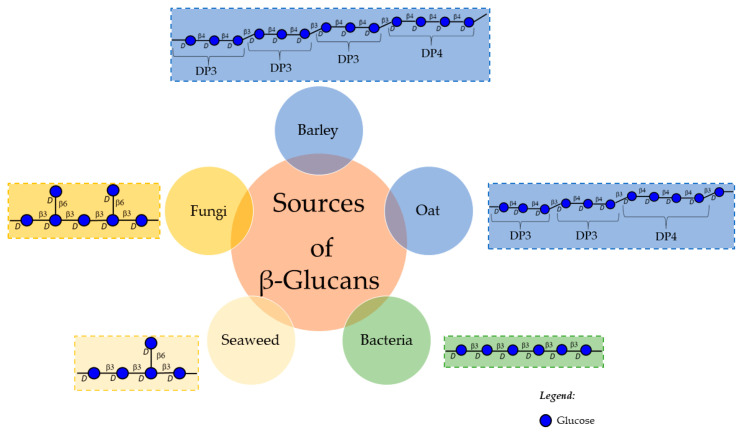
Schematic representation of β-glucans from different origins highlighting their structure diversity.

Several polysaccharides (both neutral and charged) are described to affect viscosity, such as β-glucans, galactomannans, glucomannans, arabinoxylans, pectin, alginate and chitosan (Table 1). β-Glucans are commonly found in the cell walls of cereals, fungi (mushrooms and yeast), some bacteria, and seaweed (Figure 2). 

Cereal β-glucans, such as those from oat and barley, are composed by glucose residues in (β1 → 4) glycosidic linkages, forming cellotriosyl (3 glucose residues) and cellotetraosyl (4 glucose residues) domains, intercalated by (β1 → 3) glycosidic linkages. The presence of (β1 → 3) linkages in cereal β-glucans prevents the formation of hydrogen bonds between the polymeric chains, promoting their solubility in water, contrarily to cellulose which is only composed by (β1 → 4) glucose linkages. The proportion of cellotriosyl/cellotetraosyl domains are 2 for oat and 3 for barley [20]. β-glucans with lower proportion of cellotriosyl/cellotetraosyl are more viscous. For example, a solution of 1.5% oat β-glucans with a molecular weight of 1584 kDa has a viscosity of 4500 mPa.s, whereas barley β-glucans with a similar molecular weight (1300 kDa) leads to a solution viscosity of 2600 mPa.s, measured at the same shear rate (20 s^−1^) (Table 1). Due to their viscosity, oat β-glucans have a high capacity to decrease the mobility of cholesterol solubilized in dietary micelles through intestinal lumen and this has been shown to lower serum cholesterol [21]. High viscosity oat β-glucan (2930 mPa.s) has been shown to decrease serum LDL cholesterol [22]. 

Fungi β-glucans, namely from yeast and mushrooms, are composed by (β1 → 3)-glucose residues, with side chains linked at *O*-6. The average molecular weight of yeast β-glucans, namely from *Saccharomyces cerevisiae*, was shown to be 175 kDa with a viscosity of 66 mPa.s. A decrease of molecular weight to 28 kDa leads to a viscosity of approximately half (Table 1) [23], showing that depolymerized polysaccharides still generate viscous aqueous solutions. Mushroom β-glucans, such as from *Agaricus bisporus*, with a reported average molecular weight of 181 kDa, quite resembling yeast β-glucans, present a higher viscosity, i.e., 191 mPa.s [24], than that reported for *S. cerevisiae*. The range of viscosity determined for these fungi polysaccharides is much lower than the one described for cereal β-glucans. Nevertheless, a potential decrease in cholesterol bioaccessibility can be expected, explaining the observed decrease of serum cholesterol in hyperlipidemia rat models using yeast β-glucans [25]. Laminarans (or laminarins, the old name) are also β-glucans. They occur in brown seaweeds and, similarly to fungi, are composed by linear (β1 → 3)-glucose residues with some (β1 → 6) ramifications. However, their ramifications are mainly single residues, and their reducing end can have mannitol. Laminarans low molecular weight (2–7 kDa [26]) and the occurrence of ramifications, render these polysaccharides highly soluble in water. These characteristics may explain the lack of information regarding laminarans dynamic viscosity and absence of hypocholesterolemic activity. 

β-glucans can also have origin in bacteria, such as curdlan, an exopolysaccharide derived from Alcaligenes faecalis fermentation, composed by linear (β1 → 3)-glucose residues, forming a triple helix with an average molecular weight of 1.1 MDa. This polysaccharide is not soluble in aqueous solutions. However, in alkaline medium, it can be solubilized due to the increase of flexibility of the β-glucan chains, probably due to the breakdown of hydrogen bonding. Even for polysaccharides with a molecular weight of 2.5 MDa, solubilized in a 0.5 M NaOH solution, the viscosity seems to be low (average viscosity 30 mPa.s, measured at a shear rate of 100 s^−1^) [27]. Although curdlan has been reported to have cholesterol lowering potential [28], the viscosity values determined for this polysaccharide do not seem to be relevant for its hypocholesterolemic properties. 

Galactomannans are polysaccharides with a linear chain of (β1 → 4)-linked d-mannose residues substituted with single (α1 → 6)-linked d-galactose residue. Guar gum is a galactomannan in which the ratio of mannose to galactose is 2:1. Although this polysaccharide has one of the highest molecular weights of all naturally occurring water soluble polymers, it is soluble in cold aqueous media, forming highly viscous solutions even at low concentrations. The viscosity depends on the molecular weight of the galactomannan, which ranges from 0.16 to 1.4 MDa [29]. This has been related with intermolecular chain entanglements between side chains of galactose interacting with water molecules. The viscosity of solutions containing guar gum of increasing concentrations, from 0.5% to 1.5% (Table 1), increased from 106 to 3933 mPa.s, when measured at a shear rate of 5.4 s^−1^ [30]. When measured at a shear rate of 150 s^−1^, the viscosity of a solution containing 2.0% guar gum was 1546 mPa.s [31], values that are comparable with those reported for oat β-glucans [11]. Galactomannans from locust bean gum, which have a mannose to galactose ratio of 4:1, present a higher viscosity when compared with those from guar gum (with a lower mannose to galactose ratio of 2:1) at the same shear rate and concentration (Table 1) [32]. Higher shear rates tend to decrease the measured viscosity of polysaccharides solutions, as shown in Table 1. Different authors used different shear rates, occasionally preventing a comparison of the reported polysaccharide viscosities. Due to its high viscosity, galactomannans can interfere with the diffusion of luminal cholesterol toward the epithelial cell surface [33]. 

Arabinoxylans, which are the main non-starch polysaccharide in cereals, consist of a backbone of (β1 → 4) linked xylose residues substituted with arabinose residues on the *O*-2 and/or *O*-3 position [34]. Psyllium arabinoxylans have shown to exhibit hypocholesterolemic activity mediated by their viscosity by interfering with fat and cholesterol absorption, leading to a reduction of blood cholesterol concentrations [35]. A daily consumption of 15 g of psyllium for 52 weeks was able to reduce total and LDL cholesterol by 7% and 8.1%, respectively, in overweight and obese individuals [36]. Psyllium seed husk arabinoxylans, with an Ara/Xyl ratio of 0.2–0.4 [37,38], form viscous solutions, presenting a viscosity of 519 and 15,340 mPa.s at 1.0–2.0%, respectively (Table 1) [39]. The molecular weight of these arabinoxylans is very high, ranging from 216 kDa to 1100 kDa [37,40]. This contrast with wheat bran arabinoxylans, which present an average Ara/Xyl ratio of 1 [41] and a molecular weight ranging from 83 kDa to 336 kDa [42,43,44], being less viscous (240 mPa.s) than psyllium at a concentration of 2.0%. 

Glucomannan is another polysaccharide that originate viscous solutions, usually present in konjac plants, composed of d-mannose and d-glucose linked by (β1 → 4)-glycosidic bonds at 1.6–1.4:1 Man/Glc ratio [45]. The viscosity of konjac glucomannans (KGM) with an average molecular weight of 700 kDa, measured at a shear rate of 100 s^−1^, is 1000 mPa.s (Table 1) [46], in a similar range to that of galactomannans and cereal β-glucans solutions, allowing to infer that these polysaccharides present a viscosity that can influence cholesterol bioaccessibility. Indeed, KGM has been shown to effectively reduce plasma cholesterol (11.1%) in hyperlipidemic type 2 diabetes patients when administered for 28 days (3.6 g/day) [47]. 

Chitosan is a linear polysaccharide composed by β-(1 → 4)-linked d-glucosamine and *N*-acetyl-d-glucosamine randomly distributed, being positively charged. This polysaccharide is usually obtained from the deacetylation of chitin, which is the structural element in the exoskeleton of crustaceans (such as crabs and shrimp), insects and cell walls of fungi [48]. Depending on the deacetylation degree, molecular weight, and working condition (solvent, temperature pH), solubility, and viscosity can vary extremely [49]. As expected, decrease in molecular weight of chitosan leads to a lower viscosity. When compared with β-glucans, in similar conditions, chitosan showed lower values of viscosity (Table 1). Chitosan hypocholesterolemic effect in rats was demonstrated both for low and high molecular weight chitosan, being the ones with higher deacetylation degree the most effective [50]. In humans the consumption of 3–6 g of chitosan per day allows a decrease of 6% of serum cholesterol [51]. Chitooligosaccharides have shown to decrease the total and LDL cholesterol levels in hyperlipidemic rats [52] and in humans [53]. These findings regarding chitooligosaccharides indicate that, aside from the viscosity effect, other mechanisms can be responsible for the decreased cholesterol bioaccessibility. The sequestration of BS by these oligosaccharides can demote cholesterol absorption, promoting its excretion in the feces [13]. 

Pectin is a polysaccharide with a backbone composed by d-galacturonic acid (GalA) linked by (α1 → 4)-glycosidic bonds, in which some of the carboxyl groups are esterified with methanol. Depending on the source, the main chain can contain 300–1000 galacturonic acid residues, corresponding to an average molecular weight of about 50–180 kDa [54]. The gelling properties of pectin are dependent on molecular weight and degree of methylesterification. The viscosity of pectin with the same molecular weight (322 kDa) and concentration (2%), measured with increasing shear rates, 200 and 1000 s^−1^, decreased from 60 to 40 mPa.s, indicating a pseudoplastic flow behaviour (Table 1) [55]. Experiments in both mice and humans, fed with pectins with similar viscosities but different methylation degrees (ranging from 30% to 80%), showed that plasma cholesterol levels were significantly reduced by both pectins, suggesting viscosity as the key factor behind the observed hypocholesterolemic effect [56,57]. Nevertheless, these viscosity regimes are much lower than the ones observed for other polysaccharides such as cereal β-glucans, which is reflected on the daily intake recommendations (3 g for β-glucans and 6 g for pectins) required for health claims regarding hypocholesterolemic ingredients accepted by EFSA [58,59]. 

Alginate (or alginic acid) is a copolymer of (α1 → 4)-linked β-d-mannuronate and α-l-guluronate, which can be found in the cell walls of brown algae, being a negatively charged polysaccharide. Alginate is usually linked to the gelation process that occurs in the presence of divalent cations like calcium ions, forming hydrogels. Alginates rich in guluronic acid have been shown to provide stronger gels than the ones richer in mannuronate [60]. Furthermore, the solutions containing the same concentration of alginate (1%) (Table 1) present high values of viscosity ranging from 3000 to 500 mPa.s, for shear rates of 10 s^−1^ and 100 s^−1^, respectively. Due to their gel forming properties and viscosity, this polysaccharide has cholesterol-lowering effects, which was shown in overweight male subjects [61].

## 3. Bile Salts Sequestration and Its Dependence on the Structural Diversity of Polysaccharides

BS are biological surfactants, composed by a hydrophobic and a hydrophilic surface, which are negatively charged at the intestinal pH. The most common BS are composed by three hydroxyl groups, such as cholic acid (CA) and by two hydroxyl groups the chenodeoxycholic (CDCA) and deoxycholic acid (DCA) [69]. The carboxylic group of BS can be conjugated with glycine (G) and/or taurine (T) [70]. The designation of primary BS (e.g., GCA and GCDCA, or their taurine conjugates) is used for BS synthetized in the liver, and secondary BS (e.g., GDCA and GLCA) results from their fermentation by microbiota, rendering them a more hydrophobic character. GDCA secondary BS was shown to solubilize more cholesterol than the primary BS GCA and GCDCA [18,70,71,72,73,74] and to partition to lipid membrane models with higher affinity [75]. 

Polysaccharides have been shown to interact with BS at the intestinal lumen, leading to their sequestration, which affects cholesterol bioaccessibility. The BS sequestration promotes cholesterol precipitation into crystals, which are then expelled by feces [76]. Both BS and polysaccharide structures can influence their interaction. The sequestration of BS will affect the BS enterohepatic recirculation at the ileum, which promotes the conversion of cholesterol into primary BS at the liver, recruiting LDL cholesterol from blood. The study of the interaction between BS and polysaccharides is important to gain predictive rules that can allow the development of strategies to modulate cholesterol bioaccessibility. Several features have been shown to influence this interaction, namely polysaccharide composition, their structural arrangements, such as linkages and ramifications, as well as size/molecular weight. Neutral (e.g., arabinogalactans, galactomannans, and β-glucans), positively (e.g., chitosan) and negatively charged (e.g., fucoidans) polysaccharides have been shown to sequestrate BS differently. 

The described mechanisms of interaction between polysaccharides and BS have shown that positively charged polysaccharides allow efficient binding to the negatively charged BS in the intestinal lumen. However, negatively charged or neutral polysaccharides were also shown to sequestrate BS, highlighting that other properties such as hydrophobic motifs might also be responsible for this interaction [9,10]. 

The β-glucans are polysaccharides with higher BS sequestration capacity, dependent on their structure, which can vary dependent on their origin (Figure 2). Barley and oat β-glucans have been shown to be more efficient than mushroom β-glucans [14]. When compared with other polysaccharides that originate viscous solutions, such as the neutral guar gum (Table 2), cereal β-glucans are more efficient in BS sequestration.

Furthermore, β-glucans sequestration ability towards taurocholic acid (TCA) has been shown to be two thirds lower when compared with cholestyramine, a pharmacological hypocholesterolemic cationic resin [77]. In vitro digestion of barley and oat flours, containing 12% and 16% of β-glucans, have shown more affinity to bind more hydrophobic GDCA and GCDCA than GCA [80], resulting in an increased excretion of BS [88]. On the other hand, oat β-glucans, which are more viscous than barley, were shown to have a higher retarding effect on the passage of BS across a dialysis membrane, not compatible with a sequestration mechanism [11]. Furthermore, partial hydrolysis and oxidation of barley and oat β-glucans led to a decrease in their ability to restrict the mobility of BS, attributed to its lower viscosity. However, barley β-glucans have shown to promote NMR chemical shifts in TCDCA, inferred as a direct interaction between the polysaccharide and the BS, suggesting a sequestration mechanism [79]. β-glucans with similar viscosity were shown to interact differently with BS (TCA, TDCA, and GCA) [89], showing that the diffusion of species slowed down by viscosity may also be complemented by sequestration events. 

While the neutral β-glucans have been shown to interact at a molecular level with BS, wheat arabinoxylans are not described to interact directly with BS but rather form a network that restricts their mobility [79]. NMR analysis of arabinoxylans did not show a systematic chemical shift change in BS resonances, however a decrease on the intensities of NMR BS resonances was observed, indicating that BS in solution were decreasing. It is not known if this decrease is due to sequestration or retention in the polysaccharide network. Psyllium gum, an arabinoxylan [13], also showed a correlation between the increase of viscosity of solution, due to a high polysaccharide content, and a rise in BS sequestration [89]. Arabinoxylans structure was shown to give different levels of local polymer aggregation and consequently distinct microvoids [90]. As reported for β-glucans, the interaction of arabinoxylans with hydrophobic BS is stronger [80]. 

Guar gum galactomannans have a higher capacity to bind BS than locust bean gum galactomannans [77,82]. For both polysaccharides, the binding of deoxycholate is higher than the most hydrophilic CA and TCA, being the later the one with lower affinity [91]. Galactomannan rich fractions from coffee, containing arabinogalactans, showed capacity to sequestrate GDCA. NMR analysis showed a chemical shift and a decrease in the intensity of BS resonance’s and ^13^C_4_-cholesterol peak intensity, indicative of a sequestration event and a decrease in cholesterol bioaccessibility [81]. Gum Arabic, an arabinogalactan, showed less potential as hypocholesterolemic agent than guar gum and locust bean gum, since its ability to sequestrate taurocholate is less than half [77]. Arabic gum binding of deoxycholate, although higher than cellulose, is still lower than the previous referred gums and it showed no capacity to bind cholate [91].

Chitosan and derived oligosaccharides, which are positively charged, have been shown to sequestrate BS both in vitro [13,50] and in vivo [50,51,52,53,92]. Molecular weight and deacetylation degree affect the sequestration of BS by these molecules [13]. Chitosan with a higher deacetylation degree, and thus a higher positive overall charge, has shown to bind DCA and CDCA more efficiently than the less deacetylated ones [64], suggesting that the interaction between hydrophobic groups also contributes to the sequestration events. Considering the same deacetylation degree, CA was the one showing the lowest binding capacity when compared with the two dihydroxyl BS (DCA and CDCA), highlighting the influence of BS hydrophilic/hydrophobic surface area on the interaction with polysaccharide. Chitooligosaccharides have been shown to bind dihydroxy (CDCA and DCA) BS to a greater extent than trihydroxy (sodium cholate and TCA) [13]. Calorimetric interaction of TCA BS (pKa 1.4) with chitosan was shown to occur at pH 3, below chitosan pKa (6.5) [93]. However, in the intestinal lumen, it is expected that chitosan has a less global charge character due to the higher pH, which can lead to a decrease in the electrostatic interaction. 

Pectin is also known to bind BS, although the influence of its structure on sequestration is still not elucidated. A high degree of pectin esterification, with a concomitantly lower density of negative charges, is associated with a higher binding capacity [84] due to the higher amount of hydrophobic motifs prone to interact with the BS. The BS sequestration ability of pectin has also been studied in animal models, in which the supplementation with this polysaccharide resulted in an increase of BS excretion [16], supporting the sequestration mechanism proposed in in vitro studies [84,85,86].

Fucoidans are negatively charged polysaccharides which were shown to sequestrate BS in vitro. Fucoidans are mainly composed by fucose, a C6 deoxysugar, providing hydrophobic character to these polysaccharides. This polysaccharide may be constituted by a backbone of (α1 → 3), or alternated (α1 → 3) and (α1 → 4) fucose residues with branched residues at C3 or C4 [94]. These polysaccharides are sulfated, which confer upon them negative charges able to repel each other and expose their hydrophobic domains. This effect may be responsible for the promotion of the interaction with BS [12]. Studies in mice models have shown that the consumption of fucoidans can alter the expression of enzymes related to cholesterol metabolism [95,96,97], which may be another hypocholesterolemic mechanism of this polysaccharide. 

Similarly to fucoidans, carrageenans are polysaccharides containing sulfate groups and composed by 3,6-anhydrogalactose units. Carrageenans are composed by different structural motifs, such as k-carrageenan, composed of (β1 → 3)-galactose-4-sulfate and (α1 → 4)-3,6-anhydrogalactose, ι-carrageenan which has an additional sulfate group in the O2 of the 3,6-anydrogalactose moiety, and/or β-carrageenan composed by (β1 → 3)-galactose and (α1 → 4)-3,6-anhydrogalactose. Less sulfated κ/β-carrageenans have a superior capacity to sequestrate CA than κ-carrageenan and ι/κ-carrageenan [87]. Carrageenans sequestration of TCA capacity is approximately four times less than oat and barley β-glucans [77]. 

## 4. Microbiota Bio-Transformations of Polysaccharides and Bile Salts: Hypocholesterolemic Implications

Dietary fiber is composed by nondigestible carbohydrates that pass the upper gastrointestinal tract unaffected, thus reaching the colon intact, then undergoing a complete or partial fermentation by the colonic microbiota [98,99,100]. Dietary fiber in turn modulates the intestinal microbiota, promoting health benefits by selectively stimulating the growth and/or activity of bacteria considered to have beneficial effects, such as the *Bifidobacterium* and *Lactobacillus* species [101,102,103,104]. In addition, exopolysaccharides (EPS) produced by *Bifidobacterium* sp. can also decrease cholesterol levels in obese mice [105]. An hypocholesterolemic effect was also observed *Lactobacillus paracasei M7* EPS in an in vitro model [106]. Several mechanisms have been proposed for the EPS activity, namely BS sequestration, deconjugation of BS and production of SCFA [107,108,109,110].

Over 400 species of bacteria have been identified in human feces, the two predominant phyla being the Gram-negative Bacteroidetes (e.g., *Bacteroides* and *Prevotella* genera) and Gram-positive Firmicutes (e.g., *Clostridium*, *Lactobacillus*, and *Enterococcus* genera) [111]. The fermentability of dietary fiber is highly dependent on the structural characteristics of its polysaccharides, promoting the selective growth of bacterial species. Soluble arabinoxylans contribute to the proliferation of *Bifidobacterium* and *Lactobacillus* bacterial species, while galactomannans stimulate the growth of Bacteroides [112,113]. The fermentation of dietary fiber by the gut microbiota generates short-chain fatty acids (SCFA), which are the main end product of this process and are estimated to range from 70 to 140 mM in the proximal colon [104,114]. SCFA are saturated aliphatic organic acids consisting of one (C1) to six (C6) carbons [98]. Acetate (C2), propionate (C3), and butyrate (C4) are the most abundant SCFA (90–95% of the SCFA present in the colon), being present in a molar ratio of approximately 3:1:1, respectively [115,116]. Alterations in their ratio may occur, depending on polysaccharide source and its structural composition, as well as the bacterial species involved, which can use different fermentation pathways and gut transit time [104,115,117]. Pectin, β-glucan, arabinoxylan, galactomannan, and arabinogalactan are examples of soluble dietary fiber that can positively affect colonic bacterial metabolism [100,115,118]. Pectin fermentation usually yields more acetate, while β-glucan yields more acetate and propionate than butyrate, and arabinoxylan yields more acetate and butyrate than propionate [119,120,121]. Pectins are completely degraded by gut microbiota within 6 h. An extended degradation seems to occur also by coffee galactomannans, which have been shown to be 93% degraded within 24 h. However, only 84% of arabinose residues from coffee arabinogalactans are degraded during this period [122], suggesting that the nature of sugar and the type of glycosidic linkages might hinder the polysaccharide degradation. Bacteroides can utilize alginate and its oligosaccharides [123]. The fermentation of alginate oligosaccharides modulates gut microbiota and leads to an increase in SCFA production in mice fed with a high fat diet and to a decrease in LDL-cholesterol levels [124]. A large part of the SCFA is used as a source of energy, providing about 10% of the daily energy requirement for humans [99,122,125]. About 95% of the SCFA released to medium are readily absorbed by the colonocytes, being the rest secreted in the feces [98]. Butyrate is the preferred energy source of the colonic epithelial cells, playing a major role in the regulation of cell proliferation and differentiation [115]. Once in the bloodstream, these organic acids are taken up by organs, where they can affect the lipid, glucose, and cholesterol metabolism in various tissues, with acetate and butyrate acting as precursors for cholesterol and long-chain fatty acid synthesis while propionate is mainly used for hepatic gluconeogenesis [98,125]. Despite being used as a substrate for hepatic gluconeogenesis, propionate has been shown to inhibit cholesterol synthesis in hepatic tissue [115]. 

The acetate:propionate ratio is therefore an important marker to follow lipid metabolism [104,116,119]. As this ratio depends on polysaccharides structure, they indirectly contribute to change serum lipids, having potential to control cardiovascular risk disease [104]. Studies where propionate and acetate were infused alone or in a mixture in large intestine have shown that propionate alone does not affect serum lipids, while a 3:1 ratio of acetate:propionate was able to decrease free fatty acids by 10% and reduce total and LDL-cholesterol, contrary to what was observed when acetate was infused alone [104,120]. Propionate, as well as butyrate, have been shown to stimulate the intestinal inner wall and to promote intestinal peristalsis, thus improving constipation [99,122]. The production of SCFA can also lower the pH of the large intestine, decreasing bile acid solubility, and to decrease the biotransformation of primary to secondary BS by the colonic bacterial enzyme 7α-dehydroxylase, which occurs mostly at neutral pH [99,104,117,121,122]. The bile acid pool (about 2.5–5.0 g of BS) is recycled about 4–12 times a day through enterohepatic recirculation, occurring at ileum. Although this process is very efficient, about 400–800 mg of BS escape this recirculation and are transformed by gut microbiota into secondary BS. These BS are more hydrophobic than the primary ones [19,122], being reabsorbed in the colon and transported back to the liver where they are recycled with CA and CDCA. This can have a huge impact on cholesterol solubility, increasing its bioaccessibility at intestinal lumen once they are released from liver and discharged from gall bladder [94]. On the other hand, the higher hydrophobicity of secondary BS can contribute to a favored interaction with dietary fiber, which in turn can increase its excretion. The molecular weight and/or structure of the polysaccharides play an important role in modulating the excretion of either primary or secondary bile acids. In vivo studies, where hypercholesterolemic rats were fed with barley β-glucan with different molecular weights (low: 150 kDa; medium: 530 kDa), have demonstrated that a higher level of secondary bile acid excretion is obtained in the group of rats fed with medium weight β-glucan [122].

Intestinal bacteria, such as *Bacteroides*, *Bifidobacteria*, *Clostridium*, *Enterobacter*, and *Lactobacillus*, regulate the BS metabolism through a series of enzymatic reactions, such as the deconjugation (bile salt hydrolase) and dehydroxylation (7α—dehydroxylase) of bile acids. Thus, the diversity and amount of microbiota are determinant to the composition and level of the bile acid pool [123]. Hence, in the absence of bacteria, the bile acid pool would consist of mainly primary conjugated BS [17]. Deconjugated BS have a higher pKa than the conjugated ones, and therefore a lower solubility at intestinal lumen pH. This makes them less soluble than their conjugated counterparts, and thus less reabsorbed into the gut, resulting in a higher excretion into the feces [123,124]. Deconjugation occurs mainly in the presence of *Bifidobacterium* and *Lactobacillus* strains. Thus, by increasing the colonization of these bacteria, a decrease in cholesterol solubility and an increase of fecal excretion of bile acids are observed [124,125]. As a result, more cholesterol will be used for de novo bile acid synthesis, replacing the excreted ones, lowering serum cholesterol levels [124]. 

## 5. Conclusions

Food ingredients based on polysaccharides can affect cholesterol homeostasis by several mechanisms. Viscosity may influence the diffusion of dietary mixed aggregates at the intestinal lumen, limiting cholesterol bioaccessibility, whereas interactions between polysaccharides and bile salts may reduce their emulsifying power towards cholesterol and also affect BS recirculation. Polysaccharide bio-transformations by microbiota may affect the production and ratio of different SCFA, as well as the deconjugation and conversion of primary to secondary BS, with an impact on cholesterol homeostasis. Soluble polysaccharides may be an important ingredient class to explore in the development of new hypocholesterolemic hydrophilic food matrices. The intake of these matrices after major meals, with high cholesterol content, may be more effective for the regulation of serum cholesterol levels. Positively charged short chain chitosan and chitooligosaccharides are an example of BS sequestration due to electrostatic interactions. However, non-charged polysaccharides such as galactomannans or negatively charged ones such as fucoidans are able to sequester BS possibly by hydrophobic interactions. Moreover, the biotransformation of polysaccharides by microbiota produces propionate able to inhibit the endogenous production of cholesterol at the liver. This work highlights the importance of polysaccharide structural features and their influence on the different hypocholesterolemic mechanisms able to modulate cholesterol homeostasis. Therefore, polysaccharides are relevant molecules to be considered for the development of cholesterol reducing functional foods. 

## Figures and Tables

**Table 1:** Non charged and charged polysaccharides’ viscosity dependence on concentration, molecular weight, shear rate and raw material provenience.

Charge	Name	W/V (%)	MW (kDa)	Shear-Rate (s^−1^)	Viscosity (mPas^−1^)	Food Origin	Temperature (°C)	Ref.
Non charged	β-glucans	0.5	1003	100	9.8	Barley	Room	[62]
		1.0			98			
		2.0			2.7 × 10^3^			
		1.0	nd	76	1.1 × 10^4^	Barley (commercial)	25	[63]
		1.5	1584	20	4.5 × 10^3^	Oat	37	[63]
			1300		2.6 ×10^3^	Barley		
		1.0	175	nd	65	*Saccharomyces cereviseae* (yeast)	Room	[23]
			28		39			
		10	181		1.9 × 10^2^	*Agaricus bisporus* (mushroom)		[24]
	Galactomannans	0.5	nd	5.4	1.1 × 10^2^	Guar gum	25	[64]
		1.5			3.9 × 10^3^			
		1.0	nd	200	8.0 × 10^2^	Locust bean gum	25	[32]
			nd	200	90	Guar gum		
		1.0	nd	100	5.0 × 10^2^		20-23	[65]
		1.5			1.5 × 10^3^			
		2.0			2.5 × 10^3^			
		2.0	nd	150	1.5 × 10^3^		37	[65]
	Arabinoxylans	1	nd	nd	5.2 × 10^2^	Psyllium	23	[39]
		1.5			1.1 × 10^4^			
		2.0			1.5 × 10^4^			
		1.0			1.6 × 10^2^	Wheat bran		
		1.5			2.5 × 10^2^			
		2.0			2.4 × 10^2^			
	Glucomannans	1.0	757	100	1.0 × 10^3^	Konjac	nd	[65]
		1.0	253	100	1.0 × 10^2^			
		1.0	87	100	10			
		1.0	239	50	2.9 × 10^2^		37	
			593	50	1.6 × 10^3^			
			1006	50	3.3 × 10^3^			
Positively charged	Chitosan	0.5	400	nd	92	Crab (commercial)	25	[65]
		1.0	940	nd	3.7 × 10^2^	Crab shell		[66]
			140		6.2	*Aspergillusniger* (fungi)		
			69		3.5	*Rhizopusoryzae* (fungi)		
Negatively charged	Pectins	2.0	322	200	60	Sugar beet by-products	25	[67]
				1000	40			
	Alginate	1.0	nd	1	2.0 × 10^4^	Algae	20	[60]
				10	3.0 × 10^3^			
				100	5.0 × 10^2^			
		0.04	20	200	4.0		25	[68]

**Table 2:** Bile salt sequestration by polysaccharides of different food origins.

Charge	Names	Polysaccharide Content Range	Bile Salt	Bile Salt Content Range	Food Origin	Sequestration	Ref.
Non-charged	β-glucans	0.25% (*w/v*)	TCA	2.5–20 mM	Oat	32% *	[77]
		0.25% (*w/v*)	TCA	2.5–20 mM	Barley	32% *	[77]
		0.5 mg/mL	CA (35%), DCA (35%), GCA (15%), and TCA (15%)	1.4 μmol/L	Mushroom (commercial)	75.1% *	[14]
		2.5 mg/mL	CA	1 mg/mL	Mushroom (irradiated)	17.4–48.7%	[78]
		0.083%, 0.42%, 0.83% and 1.7% *w*/*v*	TCDCA	20 mM	Barley (commercial)	Non-quantitative	[79]
		5 mg/mL	CA (35%), DCA (35%), GCA (15%) and TCA (15%)	0.14 μmol/mL	Oat	18.9–24.3%	[62]
	Arabinoxylans	25 mg/mL	GCA, GDCA and GCDCA	0.5 mM	Wheat	GCA: 0.96–1.21 GCDA: 1.08–1.41 GCDCA: 1.14–1.4 μmol BS/100 mg fiber	[80]
		0.083%, 0.42%, 0.83% and 1.7% *w/v*	TCDCA	20 mM	Wheat (commercial)	Non-quantitative	[79]
	Arabinogalactans/Galactomannans	6–18 mg/mL	GDCA	50 mM	coffee	9–46%	[81]
	Galactomannans	0.5 mg/mL	CA (35%), DCA (35%), GCA (15%) and TCA (15%)	1.4 μmol/L	Guar Gum	80% *	[14]
		4 mg/mL	TCA and TDCA	5 mM		TDCA: 31–38% TCA: 32–36%	[10]
		0.25% (*w/v*)	TCA	2.5–20 mM		25% *	[77]
		25 mg	CA	2 mg/mL		50% *	[82]
		16.5 mg/mL	CA and CDCA	133 µM/mL	Psyllium	CA: 1.2 mg/g; CDCA: 0.8 mg/g	[83]
		25 mg	CA	2 mg/mL	Locust bean gum (Commercial)	54% *	[82]
		0.25% (*w/v*)	TCA	2.5–20 mM		17% *	[77]
Positively charged	Chitosan	5, 10 and 50 mg/mL	CA, CDCA, DCA and TCA	2 mM	Losbter	Chitosan: CA: 9–17%: CDCA: 17–29%; DCA: 23–32%; TCA: 24–35%. Chitooligosaccharides: CA: 5–7%; CDCA: 2–10%; DCA: 1–6%; TCA: 1–4%	[13]
		12 mg/mL	TCA	10 mM	Sea Crab	Precipitation of 133–652 mg of cholesterol/g	[50]
		17 mg/mL	CA, DCA and CDCA	400 μmol/L	Commercial	CA: 0.2–0.6 μmol/g; DCA: 0.4–1.6 μmol/g; CDCA: 0.6–1.6 μmol/g	[64]
Negatively charged	Pectin	30 mM	GCDCA, GCA, GDCA, TDCA, TCDCA and TCA	1 mM (0.33 mM of each glyco- or tauro-conjugates)	Commercial, sugar-beet, grapefruit, oranges, lemon and lime	GCDCA: 8–15%; GCA: 6–13%; GDCA: 7–15%	[84]
		0.1 and 0.5%	TCA	2.5% *w/v*	Commercial	Non-quantitative	[85]
		0.25% (*w/v*)	TCA	2.5–20 mM	Commercial	5.5% (low-methoxy) and 9.6% (high-methoxy) of cationic resin *	[77]
		10 mg/mL	CA, DCA and CDCA	12.5 mM	Olive pomace	CA: 11–39%; DCA: 21–44%; CDCA: 17–48% of cationic resin *	[86]
	Fucoidan	1, 25 mg/mL	CA, DCA and TCA	500 μmol/L	*Laminariajaponica*	CA: 29–38%; GCA: 22–82%; TCA: 49–162% *	[12]
	Carrageenan	0.25% (*w/v*)	TCA	2.5–20 mM	Commercial	9.2% (ι-carragenan) and 10.7% (κ-carragenan) *	[77]
		0.05, 0.1 and 0.2%	TCA (46.87%), GCA (30.82%), TCDA (9.45%), GDCA (5.95%), TCDCA (2.37%), GCDCA (1.67%) and CA (0.08%)	2, 4 and 8 mM	*Chondrus armatus* (κ-carrageenan), *Tichocarpus crinitus* (κ/β-carrageenan), *Ahnfeltiopsis flabelliformis* (ι/κ-carrageenan)	κ-carrageenan: 51–66%, κ/β-carrageenan:70-74%; ι/κ-carrageenan: 33–35% *	[87]

* Percentage of relative sequestration compared to a cationic resin.

## Data Availability

Not applicable.
